# Arthroscopic therapy of rotator cuff diseases: A bibliometric study of the past 2 decades (2002–2021)

**DOI:** 10.3389/fsurg.2022.927638

**Published:** 2022-09-15

**Authors:** Hongfu Jin, Yilan Ding, Weiyang Wang, Ruixi Ye, Miao He, Wenqing Xie, Hengzhen Li, Wenfeng Xiao, Yusheng Li

**Affiliations:** ^1^ Department of Orthopedics, Xiangya Hospital, Central South University, Changsha, China; ^2^ National Clinical Research Center for Geriatric Disorders, Xiangya Hospital, Central South University, Changsha, China; ^3^ Xiangya School of Medicine, Central South University, Changsha, China

**Keywords:** arthroscopy, arthroscopic therapy, rotator cuff diseases, bibliometric analysis, web of science

## Abstract

**Purpose:**

Rotator cuff diseases, as a common cause of shoulder pain and disability, have seriously affected the patients' daily life. Rotator cuff repair techniques have been a hot topic in the arthroscopic therapy field. Our study was to use bibliometrics analysis to clarify the current status and research trends in the field of arthroscopic therapy of rotator cuff diseases.

**Methods:**

The publications relating to arthroscopic therapy of rotator cuff diseases published from 2001 to 2021 were obtained from the Web of Science Core Collection (WoSCC) database. The R software and VOSviewer software were used for the cross-sectional bibliometric and scientometric analysis.

**Results:**

A total of 4,567 publications about arthroscopic therapy of rotator cuff diseases published between 2002 and 2021 retrieved from the WoSCC database were analyzed in our study. The results showed that the United States made the largest contribution to this field. The most relevant institutions were Seoul National University, Rush University, and Hospital for Special Surgery. Stephen S Burkhart was the most relevant researcher in this field with the largest number of publications, as well as the highest H-index and G-index. The journal ARTHROSCOPY contributed the largest number of publications in the past 2 decades. Considering the H-index and G-index, ARTHROSCOPY was also the journal with the largest impact in this field.

**Conclusions:**

Arthroscopic Therapy of Rotator Cuff Diseases Related research presented a rising trend in the past 2 decades. The United States can be regarded as the leader because of its huge contributions to this field. The journal ARTHROSCOPY published the largest number of publications in this field. It can be predicted that research about advanced arthroscopic techniques and postoperative pain management of patients with rotator cuff diseases will be the next research hotspots in the following years.

## Introduction

The rotator cuff, a network structure composed of ligaments, tendons, and other connective tissues, provides stability and extremely high flexibility for the shoulder (1). However, these tissues can be injured or degenerate due to various mechanisms, including traumatic incidents, long-term repetitive activities, age-related degenerative changes of the tendon, and so on (2). Rotator cuff diseases are a common cause of shoulder pain and disability causing decreased performance in daily activities (3). Studies showed that age was a significant risk factor for rotator cuff diseases. Aging can change the physiologic characteristics and biomechanical properties of the tendon, which may result in delayed healing and an increased risk of injuries (4). Age can be considered one of the key factors in the occurrence and severity of rotator cuff diseases (5). Rotator cuff diseases are extremely prevalent among the aging population with an incidence of 30% in patients over 60 years old and 62% in those over 80 years old (6, 7). Pain and poor performance in the range of motion (ROM) of the shoulder are the main complaints from patients. With the proportion of the aging population rapidly increasing, more and more patients are suffering from rotator cuff diseases, which not only affect daily activities and work, but sports are also greatly affected. Rotator cuff pathology undergoes a progressive process, beginning with tendon impingement in the subacromial space, progressing to partial tears, full layer tears, and even cuff tear arthropathy (CTA) (8).

Interest in the field of rotator cuff diseases has constantly increased among orthopedic surgeons and researchers recently. Various rotator cuff repair treatments have been developed from physical therapy to surgical repair. The choice of rotator cuff repair is not only based on the extent of the disease and patients’ symptoms, but also the thickness, size, and morphology of the torn rotator cuff (8). Arthroscopy, as a minimally invasive orthopedic technique, is regarded as the future development trend of modern orthopedic surgery, which has been widely used in orthopedic surgery from knee diseases to other joints such as shoulder, elbow, hip, and even smaller (9). Rockwood et al. firstly performed debridement with subacromial decompression by arthroscopy for treating Rotator cuff diseases in 1995 (10). With the development of arthroscopy techniques, nowadays various arthroscopic therapy techniques have been applied in rotator cuff repair.

Bibliometric analysis is a method to clarify the current status and research trends in a specific field and identify the most valuable literature. The publications about arthroscopic therapy of rotator cuff diseases have grown annually over the past 20 years. However, no authors have conducted a comprehensive bibliometric analysis of related publications in this field in the past few years. This study aims to provide researchers with the current status and development trends of arthroscopic therapy of rotator cuff diseases research through bibliometric analysis, to identify the most valuable publications, influential journals, major researchers, and core countries in the field.

## Methods

### Database source

The cross-sectional bibliometric and scientometric analysis was conducted on February 10, 2022. The publications for arthroscopic therapy of rotator cuff diseases published from 2002 to 2021 were retrieved from the Web of Science Core Collection (WoSCC) database. WoSCC contains a worldwide variety of authoritative and high-impact academic journals covering the fields of natural sciences, engineering technology, biomedicine, social sciences, arts, and humanities. WoSCC also includes the references cited in the publications, which are indexed according to the cited author, source, and publication date. All these characteristics of WoSCC can meet the requirements for bibliometric analysis of Arthroscopic Therapy of Rotator Cuff Diseases-Related research.

### Search strategy

**Figure 1:**
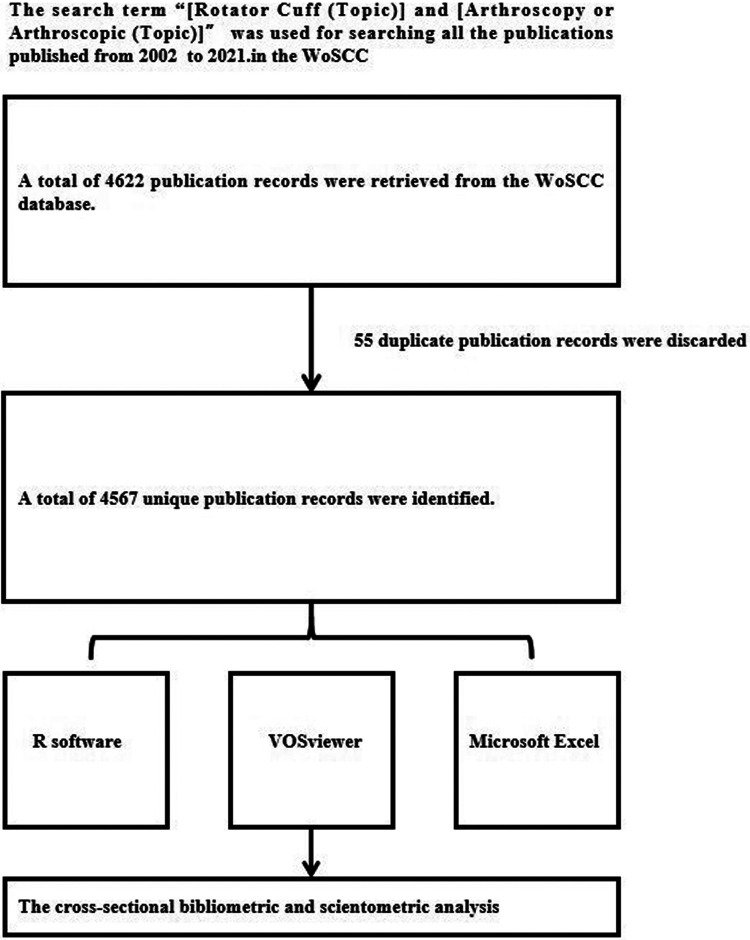
The flow chart for the “Search Strategy”: a total of 4,567 unique publication records were identified for the cross-sectional bibliometric and scientometric analysis.

We adjusted the literature published timespan from 2002 to 2021. The search terms related to rotator cuff diseases were “Rotator Cuff Disease”, “Rotator Cuff Disorder”, or “Rotator Cuff Tear”. And the search term related to arthroscopic therapy was “Arthroscopy”, “Arthroscopic Treatment”, or “Arthroscopic Therapy”. To find all the relevant publications, we used “[Rotator Cuff (Topic)] and [Arthroscopy or Arthroscopic (Topic)]” to search the literature in the WoSCC, covering all types of studies, such as Articles, Reviews, Clinical Trials, Case Reports, Editorial Materials, Case Reports, Letters, and so on. And the title, authors, countries, institutions, abstract, keywords, and references were extracted from the WoSCC, which forms raw data for our bibliometric analysis (Figure 1).

### Data analysis

The “bibliometrix” package (version 3.0.3) installed in R software (version 4.1.0) provides a web interface for comprehensive science mapping analysis (11). Microsoft Excel was used to draw the line, bar, and pie charts. Citespace (version 6.1.R2) software was used for keyword burst Analysis. And the VOSviewer (version 1.6.16) software was mainly used to conduct co-occurrence, co-citation, and co-authorship analysis (12).

### Statistical analysis

In this study, a linear regression model was established for the year and the number of publications by IBM SPSS Statistics (Version R26.0.0.0), and the *P* was set at 0.05. Most analysis results are presented in quantity and percentage (*n*, %).

## Results

### General information about arthroscopic therapy of rotator cuff diseases related publications

**Figure 2:**
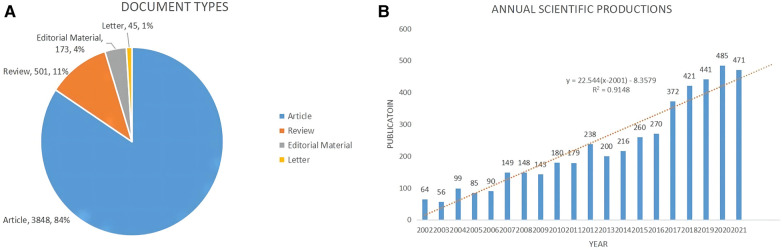
General information about arthroscopic therapy of rotator cuff diseases related publications from 2002 to 2021. (**A**) The document types of Arthroscopic Therapy of Rotator Cuff Diseases Related Publications. (**B**) The annual scientific productions of Arthroscopic Therapy of Rotator Cuff Diseases Related Publications.

Using the topic terms “Rotator Cuff and (Arthroscopic or Arthroscopy)”, there were a total of 4,622 records found in the WoSCC database. And 55 duplicate and incomplete records were removed. Finally, there were 4,567 document records (Article, 3,848, 84%; Review, 501, 11%; Editorial Material, 173, 4%; Letter, 45, 1%) published between 2002 and 2021 retrieved from the WoSCC database analyzed in this bibliometric analysis (Figure 2A). All of these document records were from 437 sources (Journals, Books, etc.). There were 11,559 authors and 66 countries contributing to this field. The number of publications relating to arthroscopic therapy of rotator cuff diseases showed a rising growth trend from 64 in 2002 to 471 in 2021 (Figure 2B). The 95% confidence interval (CI) of the number of publications in 2022 predicted by the linear regression model is (377,573) (*P* < 0.05) (Figure 2).

### Country and institution analysis

**Figure 3:**
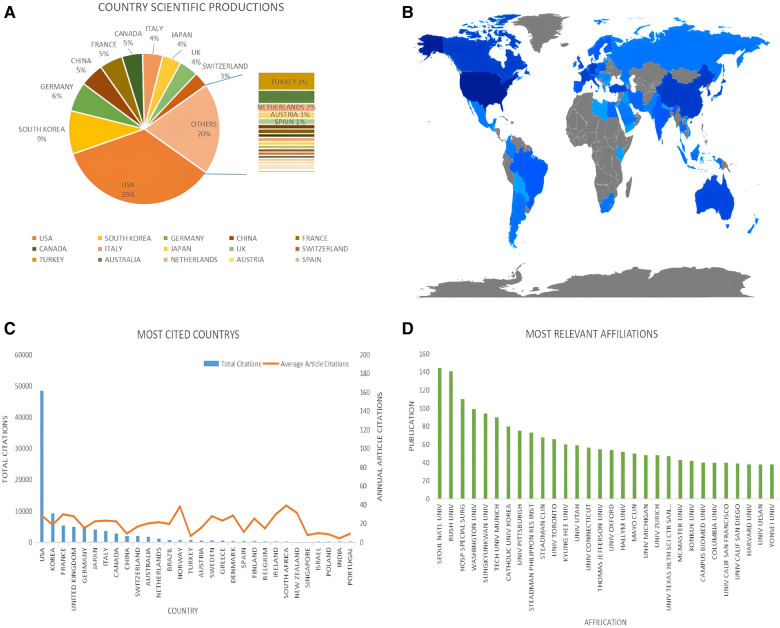
Country and institution analysis of arthroscopic therapy of rotator cuff diseases related publications from 2002 to 2021. (**A**) The production proportion of contributing countries. (**B**) Heat Map of the country's scientific productions. (**C**) The total citations and annual article citations of the top 50 countries. (**D**) The number of publications of the top 30 most relevant countries.

The characteristics of the countries and institutions of the document records were analyzed by the bibliometrix package (version 3.0.3) running in R software (version 4.1.0). 95 countries contributed to Arthroscopic Therapy of Rotator Cuff Diseases-Related publications. As Figure 3A showed, the largest number of publications was contributed by the United States (35%), followed by South Korea (9%), Germany (6%), China (5%), France (5%), Canada (5%), and Italy (4%). The national contribution map produced by the data visualization technology showed that North America, Europe, East Asia, and Oceania were the main research areas (Figure 3B: gray indicates a small number of productions, blue indicates a large number of productions, and the depth of the color increases with the number of productions).

Citation analysis can be used to identify the impact of articles, countries, or authors in specific fields. Based on the document records retrieved from the WoSCC database, Arthroscopic Therapy of Rotator Cuff Diseases-Related publications from the United States possessed the highest total citation frequencies (48,508 times), followed by Korea (9,219 times), France (5,376), the UK (4,969 times), and Germany (4,778 times). In the terms of average citation frequencies per document, the top five countries were South Africa (39 times), Norway (37.95 times), Luxembourg (37.667 times), New Zealand (31.286 times), and France (29.702 times) (Figure 3C).

There are a total of 3,254 institutions contributing to Arthroscopic Therapy of Rotator Cuff Diseases-Related research between 2002 and 2021. Seoul National University ranked first with 144 publications, followed by Rush University (141 publications), Hospital for Special Surgery (110 publications), Washington University (99 publications), and Sungkyunkwan University (94 publications). Among the top 30 relevant affiliations, there were 16 located in the United States, 8 located in Korea, 2 located in Canada, 1 located in the UK, 1 located in Germany, 1 located in Italy, and 1 located in Switzerland (Figure 3D).

### Author analysis

**Figure 4:**
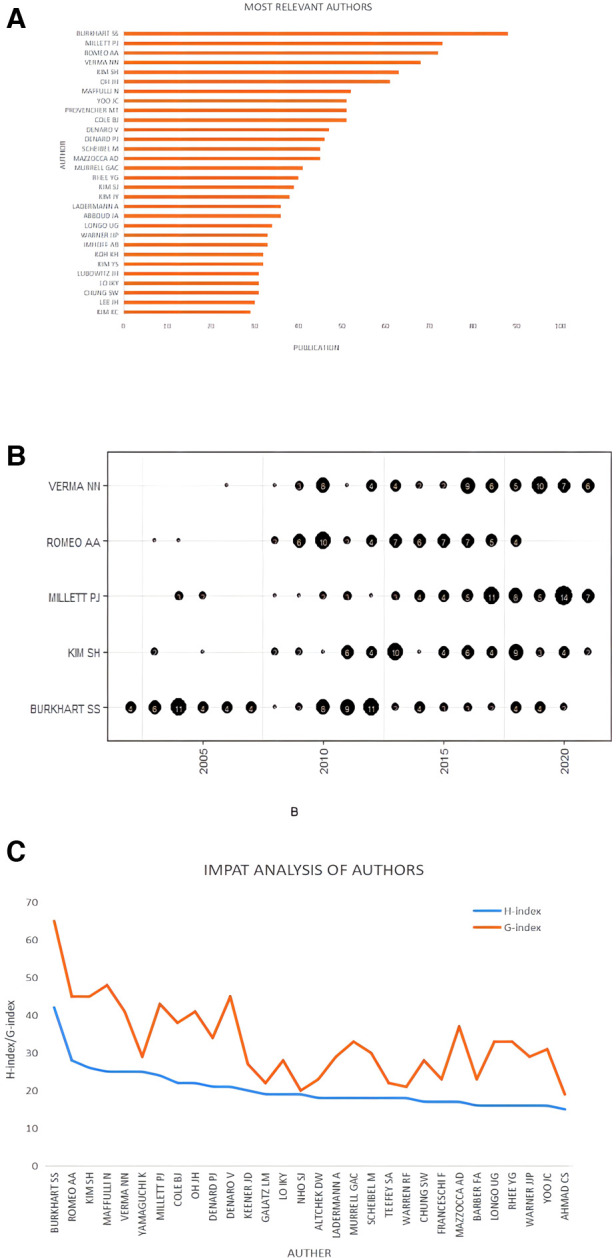
Author analysis of arthroscopic therapy of rotator cuff diseases related publications from 2002 to 2021. (**A**) The number of publications of the top 30 most relevant authors. (**B**) The analysis of the top 5 most relevant authors’ production over time. (**C**) The H-index and G-index of the top 30 most relevant authors.

A total of 11,559 researchers were involved in Arthroscopic Therapy of Rotator Cuff Diseases-Related research. The author who participated in the largest number of publications was Stephen S Burkhart (San Antonio Orthopaedic Group, Burkhart Research Institute for Orthopaedics) with 88 publications, followed by Peter J Millett (Steadman-Philippon Research Institute; Steadman Clinic) with 73 publications, Anthony A Romeo (Rothman Institute Department of Orthopaedic Surgery) with 72 publications, and Nikhil N Verma (Rush University Medical Center) with 68 publications, and Sae Hoon Kim (Department of Orthopedic Surgery, Seoul National University Hospital, Seoul National University College of Medicine) with 63 publications (Figure 4A). Figure 4B showed the top 5 authors’ production over time. Stephen S Burkhart had a high publication output during 2003–2007 and 2010–2012. Peter J Millett entered a period of high output after 2014. Anthony A Romeo, Nikhil N Verma, and Sae Hoon Kim output productions in a stable manner.

The H-index can be used to evaluate the number of academic outputs and the level of academic outputs of researchers, which can reflect the author's impact in a specific field. In terms of the H-index, the top 5 authors were Stephen S Burkhart with an H-index of 42, Anthony A Romeo with an H-index of 28, Sae Hoon Kim with an H-index of 26, Nicola Maffulli (Department of Medicine, Surgery and Dentistry, University of Salerno; School of Pharmacy and Bioengineering, Keele University Faculty of Medicine; Barts and the London School of Medicine and Dentistry, Centre for Sports and Exercise Medicine, Mile End Hospital) with an H-index of 25, and Nikhil N Verma with an H-index of 25. The G-index is a derivative of the H-index, which can also be used to reflect the impact of the authors. Generally speaking, G ≥ H and the higher the number of citations, the higher the G-index. Stephen S Burkhart also ranked first with the highest G-index of 65, followed by Nicola Maffulli with a G-index of 48, Anthony A Romeo with a G-index of 45, Sae Hoon Kim with a G-index of 45, and Vincenzo Denaro (Department of Orthopaedic and Trauma Surgery, Campus Bio-Medico University) with a G-index of 45 (Figure 4C).

### Journal analysis

**Figure 5:**
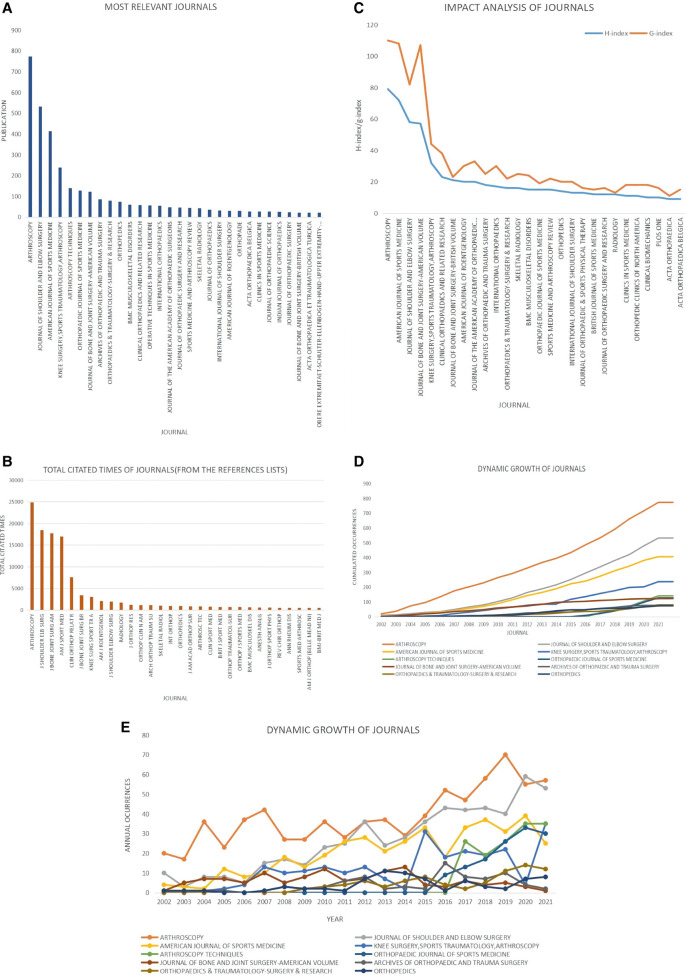
Journal analysis of arthroscopic therapy of rotator cuff diseases related publications from 2002 to 2021. (**A**) The number of publications of the top 30 most relevant journals. (**B**) The total cited times of the top 30 most relevant journals (Analysis based on the list of references). (**C**) The H-index and G-index of the top 30 most relevant journals. (**D**) The dynamic growth of top 10 journals (Analysis based on the cumulated occurrences). (**E**) The dynamic growth of top 10 journals (Analysis based on the annual occurrences).

437 Journals published Arthroscopic Therapy of Rotator Cuff Diseases-Related publications. ARTHROSCOPY ranked first, which contributed the largest number of publications with 773 papers in the past 2 decades, followed by JOURNAL OF SHOULDER AND ELBOW SURGERY with 532 papers, AMERICAN JOURNAL OF SPORTS MEDICINE with 414 papers, KNEE SURGERY, SPORTS TRAUMATOLOGY, ARTHROSCOPY with 239 papers, and ARTHROSCOPY TECHNIQUES with 141 papers (Figure 5A). Based on the analysis of references records, the top 5 highly cited journals were ARTHROSCOPY (24,917 times), JOURNAL OF SHOULDER AND ELBOW SURGERY (18,465 times), JOURNAL OF BONE AND JOINT SURGERY-AMERICAN VOLUME (17,748 times), AMERICAN JOURNAL OF SPORTS MEDICINE (16,978 times), and CLINICAL ORTHOPAEDICS AND RELATED RESEARCH (7,575 times) (Figure 5B).

The H-index and G-index of journals can reflect the impact of journals in a certain field. H-index, also called H-factor, implies that the n of papers with at least n citations. G-Index can be regarded as an improvement on the H-index, which is used to make up for the deficiency of the H-index that place a low value on highly cited publications. Figure 5C showed that ARTHROSCOPY ranked first with the highest H-index of 79, followed by AMERICAN JOURNAL OF SPORTS MEDICINE with an H-index of 72, JOURNAL OF SHOULDER AND ELBOW SURGERY with an H-index of 58, JOURNAL OF BONE AND JOINT SURGERY-AMERICAN VOLUME with an H-index of 57, KNEE SURGERY, SPORTS TRAUMATOLOGY, ARTHROSCOPY with an H-index of 32. The ranking order of journals based on the G-index was consistent with H-index, except that the JOURNAL OF BONE AND JOINT SURGERY-AMERICAN VOLUME surpassed the one in front with a high H-index of 107. Figures 5D,E presented that ARTHROSCOPY, AMERICAN JOURNAL OF SPORTS MEDICINE, and JOURNAL OF SHOULDER AND ELBOW SURGERY maintained an advantage compared with other journals in the Arthroscopic Therapy of Rotator Cuff Diseases-Related field in the past 2 decades. ARTHROSCOPY TECHNIQUES showed a rapid growth trend in this field since 2016 (Figure 5).

### Keywords analysis

**Figure 6:**
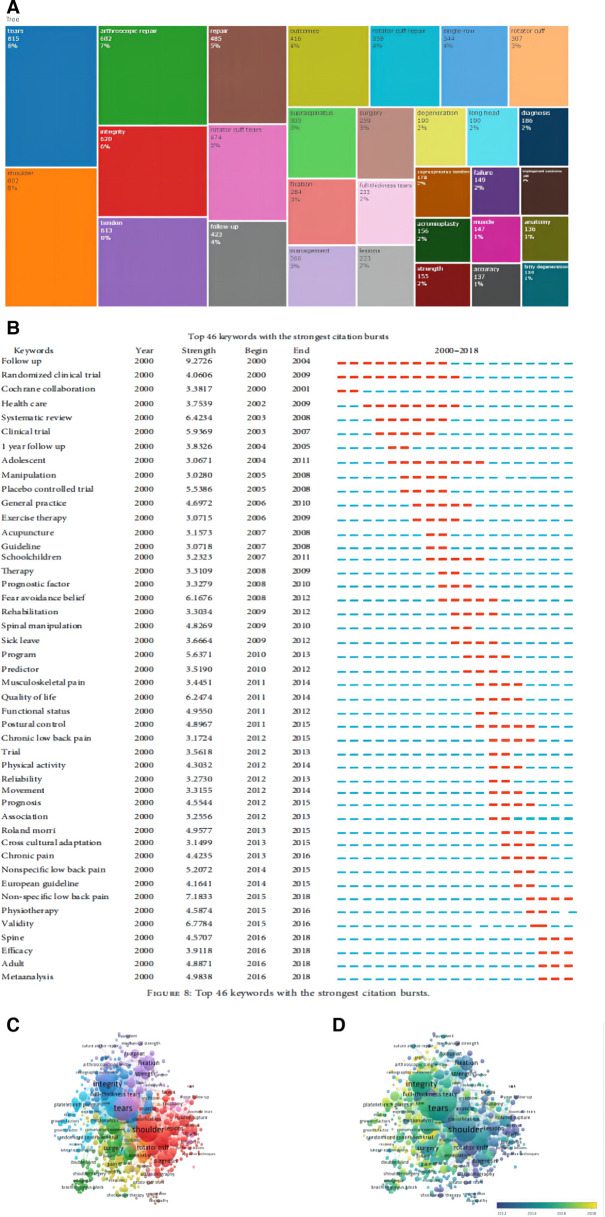
Keywords analysis of arthroscopic therapy of rotator cuff diseases related publications from 2002 to 2021. (**A**) Map of the top 30 most frequent keywords. (**B**) The dynamic growth of the top 50 most frequent keywords (Analysis based on the annual frequency). (**C**) The cluster map of the keywords subnetwork made by Vosviewer. (**D**) Distribution map of keywords according to the time of occurrence made by Vosviewer (blue-colored keywords occurrence earlier than the yellow ones).

Keywords refer to the words that can embody the central concept of an article or a publication. By analyzing the keywords retrieved from document records, we can see the high-frequency used words in a specific period, helping to insight research trends in related fields and the emergence of new hotspots. The top 10 most frequent keywords were Tear (815 times), Shoulder (802 times), Arthroscopic Repair (682 times), Integrity (620 times), Tendon (613 times), Repair (485 times), Rotator Cuff Tears (474 times), Follow-up (423 times), Outcomes (416 times), Rotator Cuff Repair (358 times) (Figure 6A). The dynamic growth of the frequency of keywords can help us to identify the burst keywords. Figure 6B showed that the keyword “Arthroscopic Repair” became hot after 2010. There was a higher frequency of the keyword “Platelet-Rich-Plasma” since 2011. The keyword “Meta-Analysis” first appeared in the field in 2014 and then began to maintain relatively steady growth. The frequency of “Sigle-Row” is higher than that of “double-row” each year. The keyword “Muscle Atrophy”, and “Fatty Degeneration” currencies in high frequency in recent 5 years.

The keyword co-occurrence analysis was conducted by Vosviewer. The cluster map of the keywords subnetwork was shown in Figure 6C. There were a total of 6 clusters identified. The biggest cluster contains 136 items, including “Shoulder”, “Rotator cuff tear”, “Lesions”, “Anatomy”, “Glenoid Labrum” and so on. From Figure 6D, We can analyze the change in research topic in this field. “Superior Capsule Reconstruction”, “Matrix Augmentation”, “lateral Epicondylitis”, “Mesenchymal Stem Cells”, and “Pain Management” were hot keywords in recent years (Figure 6).

### The top 100 most-cited articles

**Figure 7:**
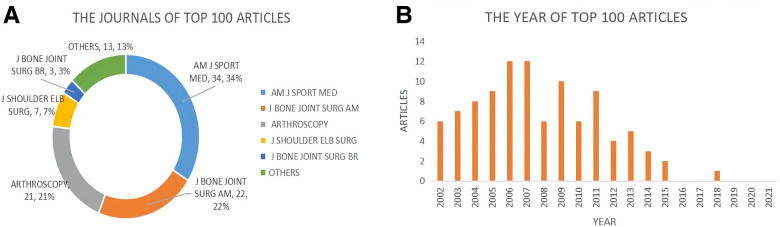
The Top 100 most-cited articles of arthroscopic therapy of rotator cuff diseases from 2002 to 2021. (**A**) The journal proportion of the top 100 most-cited articles. (**B**) The annual production of the top 100 most-cited articles.

The top 100 Most-Cited articles were mainly from AMERICAN JOURNAL OF SPORTS MEDICINE (34, 34%), JOURNAL OF BONE AND JOINT SURGERY-AMERICAN VOLUME (22, 22%), ARTHROSCOPY (21, 21%), JOURNAL OF SHOULDER AND ELBOW SURGERY (7, 7%), JOURNAL OF BONE AND JOINT SURGERY-BRITISH VOLUME (3, 3%), and others (13, 13%) (Figure 7A). Figure 7B showed that the top 100 Most-Cited articles were mainly produced between 2002 and 2018, however, there was no highly cited article produced in 2016. The article titled “The Outcome and Repair Integrity of Completely Arthroscopically Repaired Large and Massive Rotator Cuff Tears” published by Leesa M Galatz et al. in 2004 was the most highly cited article between 2002 and 2021 with a total citation of 1,263. We can learn the main information of the top 10 most highly cited articles in Table 1, there were 9 articles and 1 review.

**Table 1:** General information about the top 10 most-cited articles.

Title	Type	Author	Year	Journal	Total citations	TC per year
The Outcome and Repair Integrity of Completely Arthroscopically Repaired Large and Massive Rotator Cuff Tears	Article	GALATZ LM	2004	J BONE JOINT SURG AM	1263	66.4737
Arthroscopic repair of full-thickness tears of the supraspinatus: does the tendon really heal?	Article	BOILEAU *P*	2005	J BONE JOINT SURG AM	817	45.3889
National trends in rotator cuff repair	Article	COLVIN AC	2012	J BONE JOINT SURG AM	558	50.7273
Repair integrity and functional outcome after arthroscopic double-row rotator cuff repair. A prospective outcome study	Article	SUGAYA H	2007	J BONE JOINT SURG AM	519	32.4375
The Reverse Shoulder Prosthesis for glenohumeral arthritis associated with severe rotator cuff deficiency. A minimum two-year follow-up study of sixty patients	Article	FRANKLE M	2005	J BONE JOINT SURG AM	514	28.5556
Functional and structural outcome after arthroscopic full-thickness rotator cuff repair: single-row versus dual-row fixation	Article	SUGAYA H	2005	ARTHROSCOPY	455	25.2778
Anatomical and biomechanical mechanisms of subacromial impingement syndrome	Review	MICHENER LA	2003	CLIN BIOMECH	374	18.7
Cuff integrity after arthroscopic versus open rotator cuff repair: A prospective study	Article	BISHOP J	2006	J SHOULDER ELB SURG	368	21.6471
Clinical results of arthroscopic superior capsule reconstruction for irreparable rotator cuff tears	Article	MIHATA T	2013	ARTHROSCOPY	353	35.3

## Bibliographic coupling analysis

### Journal

**Figure 8:**
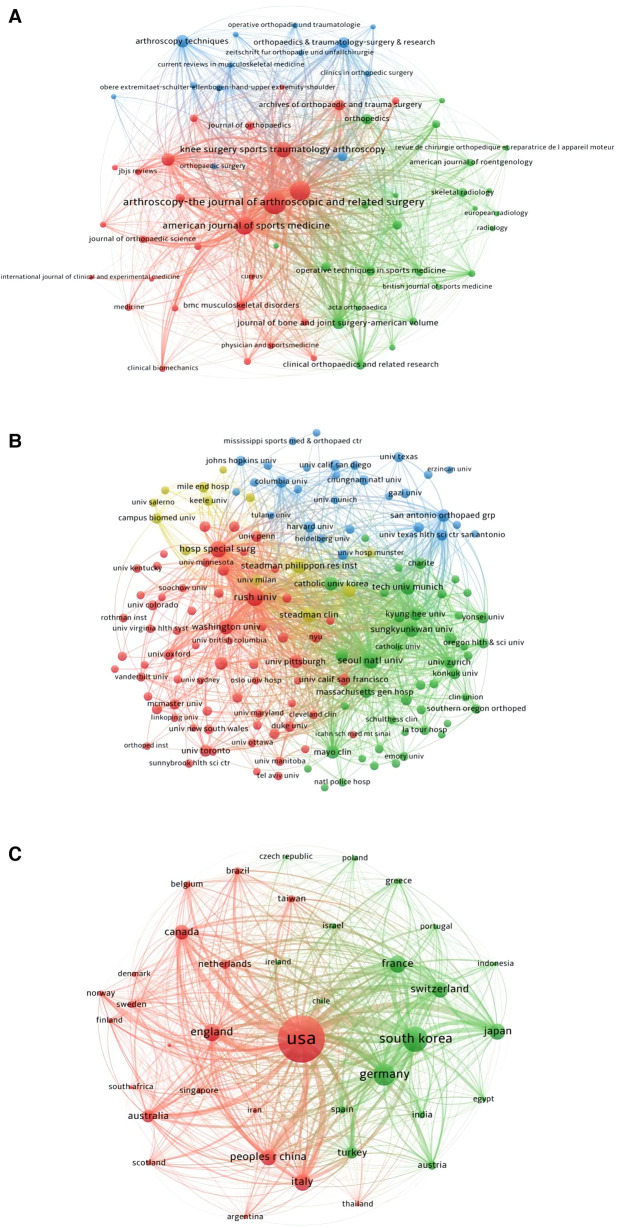
Bibliographic coupling analysis of journals, institutions, and countries (analysis based on the arthroscopic therapy of rotator cuff diseases related publications from 2002 to 2021). (**A**) Mapping of the 64 identified most relevant journals. (**B**) Mapping of the 153 identified most relevant institutions. (**C**) Mapping of the 69 identified most relevant countries.

The VOSviewer was used to conduct the bibliographic coupling analysis of journals in this study. As shown in Figure 8A, 64 journals (Minimum number of documents of a source: 10) had a similar relationship with others. The top five journals with the biggest total link strength were ARTHROSCOPY-Journal of Arthroscopic and Related Surgery (Total Link Strength = 772,328 times), AMERICAN JOURNAL OF SPORTS MEDICINE (Total Link Strength = 702,069 times), JOURNAL OF SHOULDER AND ELBOW SURGERY (Total Link Strength = 668,842 times), KNEE SURGERY, SPORTS TRAUMATOLOGY, ARTHROSCOPY (Total Link Strength = 334,332 times), and JOURNAL OF BONE AND JOINT SURGERY-AMERICAN VOLUME (Total Link Strength = 244,256 times).

### Institution

The VOSviewer was used to conduct the bibliographic coupling analysis of institutions in this study. A total of 153 institutions (Minimum number of documents of an insttution: 10) were presented in Figure 8B. The institution with the biggest total link strength was Rush University (Total Link Strength = 197,813 times), followed by Seoul National University (Total Link Strength = 178,447 times), Hospital for Special Surgery (Total Link Strength = 152,643 times), Washington University (Total Link Strength = 112,718 times), and Sungkyunkwan University (Total Link Strength = 102,964 times).

### Country

There were 69 countries (Minimum number of documents from a country: 10) presented in Figure 8C made by VOSviewer. The United States ranked first with a total link strength of 2,067,291 times, followed by South Korea (Total Link Strength = 928,037 times), Germany (Total Link Strength = 661,973 times), France (Total Link Strength = 509,490 times), and Switzerland (Total Link Strength = 410,860 times) (Figure 8).

## Co-Authorship analysis

### Author

**Figure 9:**
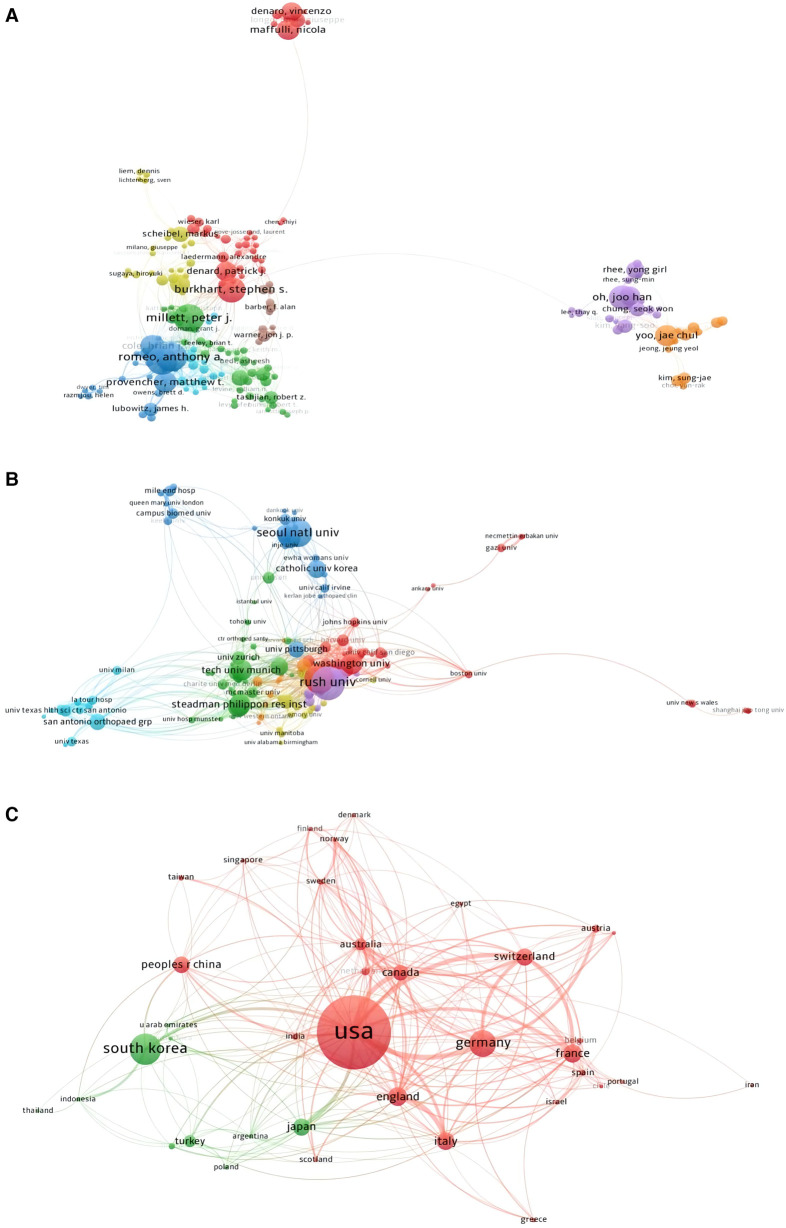
Co-authorship analysis of authors, institutions, and countries (analysis based on the arthroscopic therapy of rotator cuff diseases related publications from 2002 to 2021). (**A**) Mapping of the 174 identified the most relevant authors. (**B**) Mapping of the 148 identified the most relevant institutions. (**C**) Mapping of the 38 identified most relevant countries.

The VOSviewer was used to conduct the co-authorship analysis of authors in this study. There were a total of 174 authors (Minimum number of documents of an author: 10) were shown in Figure 9A. The largest total link strength author was Nikhil N Verma (Total Link Strength = 203 times), followed by Anthony A Romeo (Total Link Strength = 200 times), Brian J Cole (Total Link Strength = 133 times), Vincenzo Denaro (Total Link Strength = 120 times), and Peter J Millett (Total Link Strength = 112 times).

### Institution

A total of 148 institutions (Minimum number of documents of an organization: 10) were shown in Figure 9B analyzed by VOSviewer. The top five institutions with the biggest total link strengths were Steadman Philippon Research Institute (Total Link Strength = 108 times), Hospital for Special Surgery (Total Link Strength = 102 times), Rush University (Total Link Strength = 95 times), Washington University (Total Link Strength = 61 times), and Oregon Health and Science University (Total Link Strength = 61 times).

### Country

There were 38 identified countries (Minimum number of documents from a country: 10) presented in Figure 9C made by VOSviewer. The largest total link strength country was the United States (Total Link Strength = 416 times), followed by Germany (Total Link Strength = 174 times), Switzerland (Total Link Strength = 174 times), England (Total Link Strength = 170 times), and Canada (Total Link Strength = 129 times) (Figure 9).

## Co-citation analysis

### Publication

**Figure 10:**
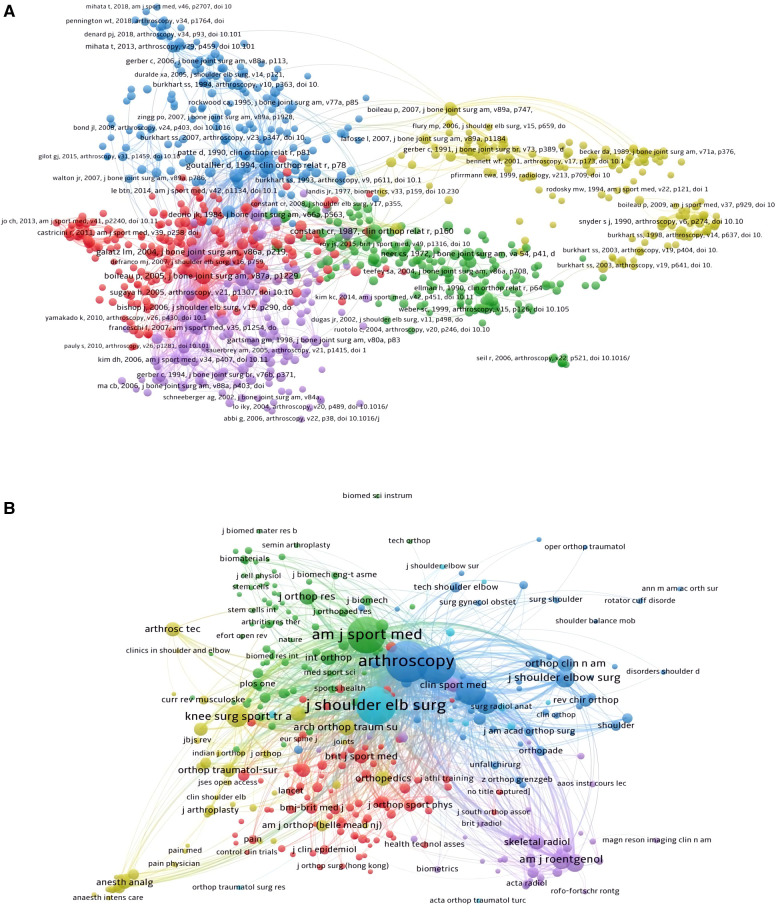
Co-citation analysis of publications and journals (analysis based on the arthroscopic therapy of rotator cuff diseases related publications from 2002 to 2021). (**A**) Mapping of the 850 identified the most relevant publications. (**B**) Mapping of the 321 identified the most relevant journals.

The co-citation analysis in our study was conducted by VOSviewer. There were a total of 850 publications (Minimum number of citations of a reference: 30) were shown in Figure 10A. The top five publications with the biggest total link strengths were as follows: “GALATZ LM, 2004, J BONE JOINT SURG AM, 10.2106/00004623-200402000-00002” (Total Link Strength = 14,845 times), “BOILEAU P, 2005, J BONE JOINT SURG AM, 10.2106/JBJS.D.02035” (Total Link Strength = 12,918 times), “GOUTALLIER D, 1994, CLIN ORTHOP RELAT R, 1994 Jul;(304):78–83.” (Total Link Strength = 12,273 times), “GERBER C, 2000, J BONE JOINT SURG AM, 10.2106/00004623-200004000-00006” (Total Link Strength = 9,116 times), and “CONSTANT CR, 1987, CLIN ORTHOP RELAT R, 1987 Jan; (214):160–4” (Total Link Strength = 9,026 times).

### Journal

There were 321 journals (Minimum number of citations of a source:30) presented in Figure 10B. The largest total link strength journal was ARTHROSCOPY (Total Link Strength = 781,934 times), followed by JOURNAL OF SHOULDER AND ELBOW SURGERY (Total Link Strength = 692,203 times), JOURNAL OF BONE AND JOINT SURGERY-AMERICAN VOLUME (Total Link Strength = 659,225 times), AMERICAN JOURNAL OF SPORTS MEDICINE (Total Link Strength = 649,381 times), and CLINICAL ORTHOPAEDICS AND RELATED RESEARCH (Total Link Strength = 303,891 times).

## Discussion

### The purpose of bibliometric analysis on arthroscopic therapy of rotator cuff diseases-related publications

Bibliometric analysis is a powerful tool to analyze the research status and predict the future trend of a field in a certain period. Shoulder pain, mainly caused by rotator cuff diseases, accounts for about 16% of musculoskeletal disorders. This disease plagues millions around the world every year, especially the elderly and active athletes (13, 14). The emergence of arthroscopy techniques has contributed greatly to the management of rotator cuff diseases. The increasing interest in the Arthroscopic Therapy of Rotator Cuff Diseases-Related field has attracted more researchers and institutions dedicated to relevant research. In our study, the current status of Arthroscopic Therapy of Rotator Cuff Diseases-Related research, contributing countries and institutions, and the impact of authors and journals were analyzed by using methods of bibliometric analysis.

### Research Status of global publications

By using data visualization software, the results can be better presented to readers. As shown in the result section, there have been a dramatically increasing number of Arthroscopic Therapy of Rotator Cuff Diseases-Related publications in recent years. A linear regression model of the year and the number of publications was established based on the available data, which forecasts that more publications will be published in the following years.

The largest number of Arthroscopic Therapy of Rotator Cuff Diseases-Related publications produced between 2002 and 2021 were contributed by the United States. The academic impact of countries and institutions can be measured by the H-index and the total number of citations. The United States was also the country with the largest academic impact around the world. In a word, the United States can be regarded as the leader in the Arthroscopic Therapy of Rotator Cuff Diseases-Related field. Korea also has a great impact in this field, when considering the total number of publications and the total number of citations. Some countries, like France, the UK, Germany, Japan, China, Italy, and Canada, have also made a great contribution to this field because of their excellent scientific productions.

The majority of the most relevant institutions were located in the United States, including Steadman Philippon Research Institute, Rush University, Hospital for Special Surgery, Washington University, and so on. Among these institutions, Rush University contributed the largest number of publications compared with other American institutions. Seoul National University in Korea produced the largest number of Arthroscopic Therapy of Rotator Cuff Diseases-Related publications around the world between 2002 and 2021. In addition, Sungkyunkwan University and the Catholic University of Korea in Korea also made great contributions to this field.

Paying attention to the academic dynamics of the most relevant researchers can help to grasp the frontier research topic in the Arthroscopic Therapy of Rotator Cuff Diseases-Related field. The top 5 authors measured by the number of publications were Stephen S Burkhart, Peter J Millett, Anthony A Romeo, Nikhil N Verma, and Sae Hoon Kim. All of them can be seen as pioneers in this field. The academic impact of researchers can be evaluated by the H-index and G-index. Stephen S Burkhart was the most outstanding researcher in this field with the largest number of publications, as well as the highest H-index and G-index.

“ARTHROSCOPY-Journal of Arthroscopic and Related Surgery” published the largest number of publications in the past 2 decades. Considering the H-index and G-index of journals, ARTHROSCOPY was also the most impactful journal. In addition, AMERICAN JOURNAL OF SPORTS MEDICINE, JOURNAL OF SHOULDER AND ELBOW SURGERY, KNEE SURGERY, SPORTS TRAUMATOLOGY, ARTHROSCOPY, and JOURNAL OF BONE AND JOINT SURGERY-AMERICAN VOLUME were excellent journals with great impact in this field. In the future, more relevant high-quality research publications may be found in these journals.

By using the method of bibliographic coupling analysis, relationships between publications in terms of journals, institutions, and countries can be investigated. The result showed that ARTHROSCOPY was the most relevant journal, Rush University was the most relevant institution, and the United States was the relevant country. The co-authorship analysis can provide information about the relationship of the items by analyzing the number of their co-authored documents, which can present the collaborative relationships among authors, institutions, and countries. Nikhil N Verma was an active researcher in this field, who was more likely to cooperate with other researchers. Steadman Philippon Research Institute established the widest cooperation relationships with other institutions around the world. And the United States had the widest cooperation with other countries. Co-citation analysis can be used to identify the relationship of items by analyzing the number of times they were cited together. The publication titled “The outcome and repair integrity of completely arthroscopically repaired large and massive rotator cuff tears” produced by Leesa M Galatz et al. in 2004 can be seen as a landmark study in the Arthroscopic Therapy of Rotator Cuff Diseases-Related field. ARTHROSCOPY was the journal with the highest cited times in this field.

### Current questions in the field of arthroscopic therapy of rotator cuff diseases

Massive rotator cuff tears refer to tears of tendon maximum diameter greater than 5 cm or tears of two or more tendons (15). Repair of massive rotator cuff tears by arthroscopic therapy is technically safe and feasible. However, there are several significant challenges for the orthopedic surgeon, such as inelastic poor-quality tendon tissue, scarring, fatty infiltration, and muscle atrophy (16). Keywords analysis showed that words like “Superior Capsule Reconstruction”, and “Fascia Autograft Patch”, indicated that multiple techniques were investigated to treat massive irreparable rotator cuff tears. Treatment strategies range from non-operative to surgical options, including debridement, partial repair, complete repair utilizing margin convergence and interval slides, tendon transfers, arthrodesis, and Reverse Total Shoulder Replacement (RTSA). Choosing treatment options based on indications is crucial to achieving the best outcomes for patients with massive rotator cuff tears (17). For patients with massive and irreparable rotator cuff tears, significant pain and dysfunction have a serious impact on the quality of life of the patients. However, there is no gold standard for non-operative treatments (18). This question is worthy of further study and exploration for researchers. Chronic degenerative rotator cuff disease is commonly recognized as an age-related, intrinsic, degenerative disease, which is also a concern for orthopedic surgeons. Studies showed that tear enlarges over time and worse pain are common in patients with chronic degenerative rotator cuff diseases. Non-operative therapeutic efficacy is frequently below expectation and surgical intervention is necessary. Those patients have limited healing potential and a high risk of recurrence after procedures (19). The severity of muscle atrophy and fatty infiltration are height correlated with the postoperative probability of tendon retear (20). In addition, rehabilitation and physical activity after arthroscopic procedures are the main concerns for both patients and surgeons. There are still controversies about the recommended postoperative rehabilitation protocol. Consistently, passive motion in the early stages is a controversial topic. Early passive motion rehabilitation can prevent postoperative stiffness, fatty infiltration, and muscle atrophy theoretically, but may also delay tendon healing. Further studies will be required to evaluate different rehabilitation protocols credibly and comprehensively.

### Future research trends of the arthroscopic therapy of rotator cuff diseases-related field

A visual knowledge map of keyword co-occurrence can help to identify the keywords that could reflect research trends in the related field. In Figure 6C, Each node represents a keyword, the size of the node is associated with the frequency of the keywords occurrence counts, the link between two nodes represents the co-occurrence of the two keywords, and different colors represent different clusters. All the keywords from 2002 to 2021 were divided into 6 clusters: Basics Anatomy and Diagnostics Related Study, Epidemiological Study including retrospective study and cohort study, Arthroscopic Technique Related Study, Postoperative Rehabilitation Related Study, Intra-Articular Injection Related Study, Rotator Cuff Diseases Combined with Other Shoulder Disorders Related Study. Figure 6D showed the relationship between occurrence years and keywords. The color changes from blue to yellow with occurrence years. Blue-colored keywords occurrence earlier than the yellow ones. Keywords with high occurrence frequency in recent years, such as “Apoptosis”, “oxidative stress”, “Inflammation”, and “Animal Model”, indicated that basic research is still popular with researchers in this field. In recent years, Yoshiaki Itoigawa et al. studied the relationship between recurrent tears after arthroscopic rotator cuff repair and superoxide-induced oxidative stress (21). Se-Young Ki et al. studied the relationship between fatty infiltration and gene expression in patients with medium rotator cuff tears (22). Sung-Min Rhee et al. studied hematologic expression in patients who underwent arthroscopic rotator cuff repair (23). These words with high occurrence frequency recently, like “Platelet Rich Plasma”, “Small-Intestine Submuc Stem Cell”, and “Mesenchymal Stem cell” indicated that intra-tendinous injection therapies were research hot topics in the related field (24). Rotator cuff diseases were challenges to the orthopedic surgeon. More and more new arthroscopic techniques were developed to improve the healing potential and initial strength of rotator cuff repair (RCR) in recent 2 decades. The Single-Row Fixations included Two-Simple Fixations, Arthroscopic Mason-Allen Fixations, and Massive Cuff Stitch. It was considered that the Double-Row repair had a higher ultimate tensile load compared with the single-row repair. Among the single-row fixations, the Massive Cuff Stitch had similar cyclic and load-to-failure characteristics to the Double-Row Fixation (25). Studies showed that double-row repair has a significantly higher rate of intact tendon healing compared to single-row repair, especially in patients with large or massive rotator cuff tears. However, there was no significant difference in clinical functional improvement. Therefore, the double-row repair should be used only in selected patients (26, 27). Suture-bridge double-row (SB-DR), a simplified double-row fixation, is considered to have superior biomechanical properties, greater footprint contact area, and pressure (28). Southern California Orthopedic Institute row (SCOI row) is a single-row arthroscopic technique, which uses triple-loaded anchors to achieve RCR. Studies showed that SCOI rows have better biomechanical properties compared to the SB-DR (29). In addition, words in Figure 6D, like “Opioid”, “Pain”, and “Acetaminophen”, showed postoperative pain management in patients undergoing RCR was popular with researchers. A fully functioning and painless shoulder joint is a vital rehabilitation target after arthroscopic therapy. Besides rehabilitation and physical activity, postoperative pain management is a vital issue and deserves further reflection. There were studies revealing that postoperative pain was associated with shoulder stiffness after arthroscopic rotator cuff repair. Therefore, strategies for early postoperative pain relief may help to decrease rate of postoperative shoulder stiffness (30). More publications on postoperative pain management may be published in the next few years.

## Limitations

By using the methods of bibliometric analysis, the most valuable publications, top journals, major researchers, and contributing countries were analyzed in the Arthroscopic Therapy of Rotator Cuff Diseases-Related field. However, there are some limitations that we must clarify. First, we used the WoSCC database as the database source, which may cause bias due to the database variation. Second, only English language publications retrieved from the WoSCC were analyzed. Language bias may exist in our study because of ignoring of Non-English language publications. The results may differ from the actual research condition because some latest published outstanding publications may be ignored because of their low cited times. Therefore, the latest published and Non-English language publications are also necessary for daily Bibliometric Analysis Related research.

## Conclusion

Arthroscopic Therapy of Rotator Cuff Diseases Related research presented a rising trend in the past 2 decades. The United States can be regarded as the leader because of its huge contributions to this field. The journal ARTHROSCOPY published the largest number of publications in this field. It can be predicted that research about advanced arthroscopic techniques and postoperative pain management of patients with rotator cuff diseases will be the next research hotspots in the following years.

## Data Availability

The original contributions presented in the study are included in the article/Supplementary Material, further inquiries can be directed to the corresponding author/s.
